# Evaluating an Insurance-Sponsored Weight Management Program With the RE-AIM Model, West Virginia, 2004-2008

**Published:** 2010-04-15

**Authors:** Christiaan G. Abildso, Sam J. Zizzi, Bill Reger-Nash

**Affiliations:** West Virginia University College of Physical Activity and Sport Sciences; West Virginia University, Morgantown, West Virginia; West Virginia University, Morgantown, West Virginia

## Abstract

**Introduction:**

Evaluations of weight management programs in real-world settings are lacking. The RE-AIM model (reach, effectiveness, adoption, implementation, maintenance) was developed to address this deficiency. Our primary objective was to evaluate a 12-week insurance-sponsored weight management intervention by using the RE-AIM model, including short-term and long-term individual outcomes and setting-level implementation factors. Our secondary objective was to critique the RE-AIM model and its revised calculation methods.

**Methods:**

We created operational definitions for components of the 5 RE-AIM indices and used standardized effect size values from various statistical procedures to measure multiple components or outcomes within each index. We used χ^2^ analysis to compare categorical variables and repeated-measures analysis of variance to assess the magnitude of outcome changes over time.

**Results:**

On the basis of data for 1,952 participants and surveys completed by administrators at 23 sites, RE-AIM indices ranging from 0 to 100 revealed low program reach and adoption (5.4 and 8.8, respectively), moderate effectiveness (43.8), high implementation (91.4), low to moderate individual maintenance (21.2), and moderate to high site maintenance (77.8). Median (interquartile range) weight loss was 13 lb (6.5-21.4 lb) among participants who completed phase I (12 weeks; 76.5%) and 15 lb (6.1-30.3 lb) among those who completed phase II (1 year; 45.7%).

**Conclusions:**

This program had a significant, positive effect on participants and has been sustainable but needs to be expanded for more public health benefit. The RE-AIM model provided a useful framework to determine program strengths and weaknesses and to present them to the insurance agency and public health decision makers.

## Introduction

Regardless of the success of diet and physical activity programs in controlled research settings (1-6), if large-scale programs are not effective or easily implemented in real-world settings they fall short of their intended purpose. In the *Strategic Plan for NIH Obesity Research*, the National Institutes of Health Obesity Research Task Force (7) outlined a national strategy for obesity research emphasizing the use of translational research. However, evaluation of the translation of effective programs to real-world settings is lacking (8).

The RE-AIM model (9) was developed as a tool to evaluate health promotion programs along 5 dimensions: reach, effectiveness, adoption, implementation, and maintenance. Reach refers to the percentage of potential participants who enroll and their representativeness of the target population; effectiveness, to the changes in participant outcomes during the program; adoption, to the number and representativeness of sites willing to conduct the program; implementation, to site adherence to program protocols; and maintenance, to individual outcomes after final intervention contact and program continuation at sites over the long term (8-10).

The RE-AIM model has been used to evaluate a limited number of programs, including 14 WISEWOMAN cardiovascular screening interventions in North Carolina (11), a church-based physical activity intervention in South Carolina (12), and 2 diabetes self-management interventions (13). To our knowledge, no evaluation of a large-scale weight management program using RE-AIM methods has been published, but studies have shown the model is applicable to a variety of health behaviors and programs. Additionally, researchers have recommended revising RE-AIM index calculation methods to reduce the likelihood of difficult-to-interpret negative values (13).

Our primary objective was to use RE-AIM to evaluate a multisite, insurance-sponsored weight management program that produced positive short-term physical and psychosocial changes in participants in a university laboratory setting. Our secondary objective was to critique the RE-AIM model's usefulness as an evaluation tool for health promotion programs. We analyzed 3- and 12-month participant outcomes and site-level program implementation information.

## Methods

This study evaluated a weight management program that has been offered since 2004 by a large public employees' insurance agency in West Virginia as a benefit to its members. Obese insured members (body mass index [BMI] ≥30 kg/m^2^) are eligible for the program, as are overweight members (BMI 25.0-29.9 kg/m^2^) who also report being treated for hypertension, diabetes, heart disease, metabolic syndrome, or sleep apnea. Participants enroll in the program by calling the insurance agency to be screened for height, weight, and health information and must have written approval from their primary care physician.

The insurance agency also screens site providers to ensure the site has adequate safety devices, exercise equipment, and staffing to accommodate high-risk participants. At the time of this study, the insurance agency had approved 31 fitness facilities ("sites") to accept participants: 2 housed in university cardiac rehabilitation/exercise physiology laboratories, 2 in physical therapy facilities, 5 in hospitals, and 22 private wellness/fitness centers. Searches of available print (14) and online (www.wvha.com, www.yellowpages.com) resources yielded 352 of these types of facilities in West Virginia. Each approved site is required to designate an administrator to act as liaison with the insurance agency, handle participant contact, coordinate site services, and enter participant data into a database.

The weight management program evaluated in this study is a 1-year benefit, during which weight loss is promoted by using a combination of behavior therapy (eg, food logs), individualized site-based exercise programming, and individualized diet therapy. Case management nurses track participant progress on the basis of monthly entry of the following body measurements by the sites into a Web-based database: weight and BMI, systolic and diastolic blood pressure, body fat percentage, waist circumference, and number of site visits. Each site determines its measurement protocols on the basis of available instruments and staff training. Sites are compensated via monthly member copayments and insurance agency payments for the provision of on-site services in 2 phases.

### Phase I (months 1-3)

Services provided during phase I include 1) access to the exercise site, 2) a 60-minute fitness evaluation and exercise program designed by an exercise physiologist, 3) a 60-minute evaluation and nutrition program designed by a registered dietitian, 4) monthly 30-minute exercise sessions with a personal trainer, and 5) 60-minute follow-up reassessments by the exercise physiologist and registered dietitian at the end of month 3. Participants may be removed from the program if they fail to exercise at the site at least twice per week; turn in food logs periodically; attend appointments with the exercise physiologist, registered dietitian, or personal trainer; or have body measurements taken by site staff monthly. Phase I is considered the "intervention" period for this evaluation. If participants meet the behavioral requirements and lose 12 lb (or show substantial improvements in other physical measures) they may continue to phase II.

### Phase II (months 4-12)

Phase II spans the remainder of the first year (9 months). This phase is considered the postintervention period for the current evaluation because it is more like a private gym membership than a weight-loss intervention; it provides access to the facility and 1 personal training session per month. Participants are expected to continue exercising at the facility twice per week and have physical measurements and visit data submitted monthly by the sites to maintain the benefit.

The insurance agency dictates what services are offered, but not how the services are implemented. The use of relevant weight management guidelines of the American College of Sports Medicine (ACSM) (15) and the American Dietetic Association (16) is suggested. For example, the exercise physiologist may choose from fitness testing methods recommended by ACSM (eg, 6-minute walk test, 1-mile walk test) and the registered dietitian may use his or her discretion in diet composition or outcome goals.

### Data sources

We used archival participant data and site administrator surveys to evaluate the weight management program. The insurance agency granted us access to an online password-protected database containing demographic and physical measurement data of all program participants. The university's institutional review board approved the study for the protection of human participants. We downloaded, verified, de-identified, and imported archival data of all participants into SPSS version 14.0 (SPSS, Inc, Chicago, Illinois).

To gather site-level program evaluation data, we recruited site administrators by mail, e-mail, and telephone (17) to complete an online survey. The survey assessed 1) site staffing, equipment, and facilities; 2) program implementation methods; and 3) barriers to program implementation. Section 2 of the survey was relevant to this study; it included items that ascertained the procedures (open-ended) or frequency (categorical) of conducting 14 program components during phase I as recommended or required by the insurance agency: measuring weight, waist circumference, body fat percentage, and blood pressure; providing individualized nutrition and fitness assessments, nutrition classes, fitness plan, one-on-one personal training, and follow-up nutrition and fitness assessments; tracking participant attendance; and reviewing home exercise and food logs or journals. Responses were recoded to dichotomous variables (yes/no) and the sum of yes responses tallied to represent the number of program components implemented.

### Statistical analyses of RE-AIM indices

**Table:** Box. RE-AIM Components and Operational Definitions

**RE-AIM Component**	**Definition**
Individual participation rate (IPR)	The percentage of eligible members of the insurance agency who participated in the weight management program
Demographic representativeness (DR)	How well participants represented the population eligible for the program
Individual completion rate (ICR)	The percentage of participants who completed each of the phases of the weight management program
Outcomes (O)	Weight change experienced by completers of each phase of the weight management program
Differential impact (DI)	A measure of differential changes in weight experienced among phase I and II completers by sex and age
Setting participation rate (SPR)	The percentage of eligible cardiac rehabilitation centers, physical therapy clinics, wellness/fitness centers, and health clubs in the state that participated in the weight management program
Component implementation rate (CIR)	The percentage of program protocols implemented at each site
Setting continuation rate (SCR)	The percentage of program sites that have elected to continue conducting the program for more than 1 year

Presenting results using the RE-AIM model first requires creating operational definitions for multiple components in each of the 5 RE-AIM dimensions (10) (Box). These components were used to calculate RE-AIM indices (Appendix). Index calculations used standardized effect size (18) values from various statistical procedures to measure multiple components or outcomes in each index. We used χ^2^ analysis to compare categorical variables and repeated measures analysis of variance (ANOVA) to assess the magnitude of outcome changes over time. In the indices, "positive" effects were reduced by differential or "negative" effects (eg, program attrition). This index calculation method has resulted in negative values in prior studies (13) for 2 reasons. First, in calculating index values, differential effects have been subtracted directly from outcome effects, overstating the "negative" program effect. To limit this potential distortion, we calculated a "proportion of positive effect not explained by differential effects" by first subtracting the differential ("negative") effect size from 1 and then multiplying the difference by the "positive" effect (Appendix). Second, a negative index value may still result if the effect size is more than 1. We resolved this challenge by using effect size measures whose upper limit was 1.

Effect size measures (and interpretation) included the squared Cramer phi (φ_c_^2^) for χ^2^ tests (small [φ_c_^2^ ≈ .01], moderate [φ_c_^2^ ≈ .09], or large [φ_c_^2^ > .25]) and the squared curvilinear correlation coefficient (partial eta squared; η^2^) for ANOVA (small [η^2^ ≈ .01], moderate [η^2^ ≈ .06], or large [η^2^ > .14]) (18,19). All raw index scores ranging from 0 to 1 were multiplied by 100 for summary index values of 0 to 100. Missing data were excluded from analyses, which were conducted by using SPSS version 14.0.

## Results

The data for 1,952 participants from 31 sites were exported, and administrators from 27 of 31 active sites (87.1% response rate) completed online surveys. Responses from 4 new sites whose participants had not yet completed phase I of the program were excluded from analysis, resulting in 23 completed site administrator surveys. The distribution of most of the participants' measurement data was skewed, and data are presented as median (interquartile range) unless otherwise noted.

### Individual-level impact ([*reach* * *effectiveness*] / 100)

A total of 60,041 adult members were covered by the insurance agency (N. Henderson, health promotions director, written communication, January 2008). By using the state obesity (31%) and overweight (36%) prevalence rates (20), and national prevalence of overweight adults with at least 1 comorbid condition (66%) (21), we estimated that 32,878 insurance members may have been eligible for the program. At the time of data collection, 1,952 members had participated (5.9% individual participation rate) (Table 1).

Baseline physical measurements for men and women are presented in Table 2. A significantly larger percentage of participants than the eligible population were women (80.0% vs 54.1%; *P* < .001, φ_c_^2 ^= .073), and more of them were aged 45-54.9 years and fewer of them were aged 65 years or older (*P* < .001, φ_c_^2^ = .113), yielding a *reach* value (*R*) of 5.4 (Table 1).

Phase I outcome changes are presented in Table 3. Of 1,647 participants who could have completed phase I (those who had started the program at least 12 weeks before data collection), 76.5% had done so. Fewer women (74.8%) than men (83.7%) completed phase I (*P* = .001, φ_c_^2 ^= .007), and completion rates tended to rise with age. Participants who completed phase I had significant weight loss (13 lb [6.5-21.4 lb]; *P* < .001, η^2^ = .592), and men lost significantly more weight than women (*P* < .001, η^2^ = .050). Differences in weight loss were also revealed among age groups *(P* = .003, η^2^= .014). These components produced an *effectiveness* value (*E) *of 43.8 and an individual-level impact ([*R* * *E*] / 100) of 2.4 (Table 1).

### Setting-level impact ([adoption * implementation] / 100)

There were 31 active weight management program sites out of a total of 352 potential sites in West Virginia at the time of data collection, resulting in an *adoption* value (*A*) of 8.8 (Table 1). Site survey responses (N = 23) showed that sites implemented a mean of 12.8 (standard deviation [SD] 1.0) of 14 program components during phase I. All sites measured weight, waist circumference, body fat percentage, and blood pressure; tracked attendance; and provided initial and follow-up nutrition assessments with a registered dietitian, nutrition classes designed by the registered dietitian, and an individualized exercise prescription. The least frequently implemented component, by 15 sites (65%), was having participants maintain home exercise logs. Implementation survey data yielded an *implementation* value (*I*) of 91.4 and a setting-level impact ([A * I] / 100) of 8.0 (Table 1).

### Long-term maintenance ([individual maintenance * setting maintenance] / 100)

Phase II outcome changes are presented in Table 4. Of 762 participants who could have completed phase II (those who had started the program at least 1 year before data collection), 348 (45.7%) had done so (*ICR_PhII_ = .*457) (Table 1). Excluding the small samples of participants aged 18-24.9 years (n = 2) and 65 years or older (n = 9), completion rates tended to increase with age and be higher in men in each age group. Participants who completed phase II achieved significant weight loss from baseline (15 lb [6.1-30.3 lb]; *P* < .001, η^2^ = .467), shedding 6.7% (2.7%-12.7%) of baseline body weight. Weight loss was similar among phase II completers of different age groups *(P* = .61, η^2^ = .011) and between sexes *(P* = .21, η^2^ = .005). These values combined to yield an *individual maintenance* value *(M_I_*) of 21.2 (Table 1).

Four of 18 sites (22%) that had been approved to accept participants at least 1 year before this study had stopped or had been disallowed to continue accepting participants, resulting in a *setting maintenance* value (M_S_) of 77.8 and a long-term maintenance value ([M_I_ * M_S_] / 100) of 16.5 (Table 1).

## Discussion

We achieved our primary objective of using RE-AIM to evaluate a weight management program. We found moderate program effectiveness and high implementation, suggesting the program has been beneficial for participants and can be implemented in a variety of settings. We found low program reach and adoption, suggesting the program could be improved by recruiting new participants and sites. Recruitment may prove difficult, however, because participants must be highly motivated to enroll in the program, and sites are required to offer services by highly trained personnel often unavailable in rural areas of West Virginia. In the long term, site maintenance was high, but individual maintenance was fairly low, indicating the program is sustainable but the services of phase II may need to be revised to improve participant outcomes. The summary results suggest this weight management program has potential to be expanded for more translation and public health benefit and should be considered a viable model for other public and private insurers.

Individual short-term and long-term outcome changes are comparable with those of other behavioral programs and clinical trials. Short-term attrition from this weight management program (23.5%) was slightly higher than is commonly seen in behavioral programs of similar length (10%-15%), though the median weekly weight loss in this program is comparable (1.23 lb vs 1.1 lb) (22). This program also compares favorably with clinical trials of similar length, which average 85%-95% completion rates and approximately 1 lb of weekly weight loss (1,5).

Long-term individual results also compare favorably with other behavioral programs and clinical trials. Participants who completed phase II (n = 348) lost a mean of 20.9 (SD, 22.3) lb from baseline, with some recidivism. Slightly more than half of phase II completers (51.5%) maintained phase I weight loss or continued losing weight in phase II. In comparison, approximately 60% to 70% of weight loss is maintained for a year after treatment in other short-term behavioral interventions (22). One-year results indicate more average weight losses but lower completion rates than randomized control trials of similar length (1,5).

We also achieved the secondary objective of critiquing the RE-AIM model and revised index calculations. The strength of the RE-AIM model is its ability to quantify for decision makers a program's strengths and weaknesses. Comparison with other health promotion program evaluations that used RE-AIM is limited at this point in the model's refinement because no 2 studies have used the same index calculation methods. We believe this study advances the RE-AIM model by 1) providing methods for assessing long-term maintenance at the individual and site levels and 2) addressing 2 methodologic concerns with existing index calculation methods (ie, negative index values and effect sizes with varying maximum values). The revised methods in this study produced positive *R* and *M_I_* values, whereas previously used methods (10) would have yielded negative values.

### Limitations

The study is limited in a number of ways. Multiple sources of measurement error may have affected the data, including 1) lack of standardized procedures and instruments for measuring health outcomes, 2) missing outcome data, and 3) social desirability of sites when entering participant data and completing survey items. Potential error was addressed in multiple ways. Trained exercise professionals took measurements using accepted professional standards of practice, the insurance agency periodically audited site data, we contacted sites to collect missing data, and all survey recruiting material stressed the informative (not punitive) nature of the study and independence of the investigators from the insurance agency. Measurement error would be more important in a small clinical trial assessing an intervention's efficacy than in this study with its large sample and focus on standardized effect sizes.

### Conclusions

Numerous questions remain to be answered before the public health effect of this and other weight management programs can be better understood. This program had a significant positive effect on participants and was sustainable, but needs to be expanded. The RE-AIM model provided a framework through which the translation of this program could be evaluated and presented to public health decision makers. In fact, the summary data of this project were presented to the insurance agency and used as evidence for changing the program benefit to address the low reach, adoption, and long-term individual maintenance. We encourage continued study of RE-AIM index calculations and application of the model to evaluate obesity treatment programs. It may be beneficial to add qualitative process data to these outcomes to work toward developing a set of best practices in behavioral weight management (11,12). Additionally, because insurance agencies often provide benefits for multiple weight management modalities (eg, behavioral, surgical, pharmacologic), RE-AIM methods should be used to evaluate multiple modalities concurrently to allow for side-by-side comparison and facilitate decision making about resource allocation. These evaluations will need to link program costs, insurance claims, individual outcomes, and future cost savings. Such analyses may affect the benefit and incentive structure of this insurer and others to improve the translation of knowledge regarding clinical obesity treatment into innovative programming that can be widely implemented.

## Figures and Tables

**Table 1:** RE-AIM Model Component and Index Values Used to Evaluate a Weight Management Program, West Virginia, 2004

**Component**	RE-AIM Index	**Value** a
Individual participation rate (IPR)		IPR = 1,952 / 60,041 * (.31 + [.36 * .66]) IPR = .059
Demographic representativeness (DR)		DR = (.073 + .113) / 2 DR = .093
	*Reach (R)*	R = (.059 * [1 – .093]) * 100 *R = 5.4*
Phase I individual completion rate (ICR_PhI_)	* *	ICR_PhI_ = 1,260 / 1,647 ICR_PhI_ = .765
Phase I outcome (O_PhI_)	* *	O_PhI_ = .592
Phase I differential impact (DI_PhI_)	* *	DI_PhI_ = (.050 + .014) / 2 DI_PhI_ = .032
	*Effectiveness (E)*	E = (.765 * .592 * [1 - .032]) * 100 *E = 43.8*
Setting participation rate (SPR)	* *	SPR = 31 / 352 SPR = .088
	*Adoption (A)*	A = (.088 * 100) = 8.8
Component implementation rate (CIR)	* *	CIR = 12.8 / 14 CIR = .914
	*Implementation (I)*	I = (.914 * 100) = 91.4
Phase II individual completion rate (ICR_PhII_)	* *	ICR_PhII_ = 348 / 762 ICR_PhII_ = .457
Phase II outcome (O_PhII_)	* *	O_PhII_ = .467
Phase II differential impact (DI_PhII_)	* *	DI_PhII_ = (.005 + .011) / 2 DI_PhII_ = .008
	*Individual maintenance (M_I_)*	M_I_ = (.457 * .467 * [1 - .008]) * 100 *M_I_ = 21.2*
Setting continuation rate (SCR)	* *	SCR = 14 / 18 SCR = .778
	*Setting maintenance (M_S_)*	M_S_ = (.778 * 100) = 77.8

Abbreviation: RE-AIM, reach, effectiveness, adoption, implementation, maintenance.

a Methods of calculating the values are described in the Appendix.

**Table 2:** Baseline Measurements of Participants, Weight Management Program, West Virginia, 2004

**Measurement**	Women (n = 1,561)		Men (n = 391)	

	No.^a^	Median (IQR)^b^	No.^a^	Median (IQR)^b^
Age, y	1,555	49.7 (41.7 to 55.8)	391	51.5 (43.0 to 57.8)
Height, in	1,557	64.0 (63.0 to 66.0)	390	71.0 (69.0 to 73.0)
Weight, lb	1,428	220.8 (192.0 to 258.4)	354	269.9 (232.7 to 333.8)
BMI, kg/m^2^	1,426	38.5 (32.8 to 43.1)	353	37.6 (32.8 to 44.8)
Systolic blood pressure, mm Hg	1,359	126.0 (118.0 to 136.0)	330	130.0 (120.0 to 140.0)
Diastolic blood pressure, mm Hg	1,359	80.5 (9.9)^c^	330	82.1 (9.8)^c^
Body fat, %	1,193	45.9 (41.9 to 49.4)	302	36.1 (31.6 to 42.8)
Waist, in	873	43.0 (38.6 to 47.5)	226	47.4 (43.5 to 53.0)

Abbreviations: IQR, interquartile range; BMI, body mass index.

a Sample sizes vary because of missing data for some measurements.

b Median and IQR are presented for data with skewed distributions unless otherwise noted.

c Measurement has a normal distribution and is presented as mean (standard deviation).

**Table 3:** 12-Week Measurements and Changes From Baseline of Phase I Completers, Weight Management Program, West Virginia, 2004^a^

**Measurement**	Phase I Completers (n = 1,260, 76.5%)			

	End Phase I		Change From Baseline	

	No.^b^	Median (IQR)^c^	No.^b^	Median (IQR)^c^
**Women (n = 993; 74.8% completion rate)**				
Weight, lb	971	205.4 (179.0 to 242.5)	970	−12.0 (−19.8 to −6.0)
Weight, %	NA	NA	970	−5.5 (−8.7 to −2.8)
BMI, kg/m^2^	971	34.6 (30.9 to 40.0)	970	−2.1 (−3.3 to −1.0)
Systolic blood pressure, mm Hg	769	122.0 (113.0 to 130.0)	764	−5.0 (16.1)^d^
Diastolic blood pressure, mm Hg	769	77.1 (8.9)^d^	764	−3.4 (10.5)^d^
Body fat, %	804	43.5 (39.7 to 47.9)	740	−2.0 (3.2)^d^
Waist, in	690	39.9 (36.0 to 44.0)	514	−2.5 (−4.1 to −1.5)
**Men (n = 267; 83.7% completion rate)**				
Weight, lb	263	251.5 (219.5 to 305.5)	263	−16.9 (−29.3 to −8.0)
Weight, %	NA	NA	263	−6.1 (−10.2 to −3.0)
BMI, kg/m^2^	263	35.1 (30.8 to 41.3)	262	−2.3 (−3.9 to −1.1)
Systolic blood pressure, mm Hg	223	124.0 (117.0 to 132.0)	213	−7.3 (13.6)^d^
Diastolic blood pressure, mm Hg	223	76.9 (8.7)^d^	213	−4.6 (9.7)^d^
Body fat, %	214	33.0 (29.1 to 39.0)	199	−3.6 (4.9)^d^
Waist, in	199	45.0 (41.0 to 50.8)	148	−2.5 (−4.4 to −1.3)

Abbreviations: IQR, interquartile range; NA, not applicable; BMI, body mass index.

a 1,647 participants could have completed phase I.

b Sample sizes vary because of missing data for some measurements; negative change values indicate improvements from baseline to the end of phase I (12 weeks).

c Median and IQR are presented for data with skewed distributions unless otherwise noted.

d Measurement has a normal distribution and is presented as mean (standard deviation).

**Table 4:** One-Year Measurements and Changes From Baseline of Phase II Completers, Weight Management Program, West Virginia, 2004^a^

**Measurements**	Phase II Completers (n = 348, 45.7%)			

	End Phase II		Change From Baseline	

	n^b^	Median (IQR)^c^	n^b^	Median (IQR)^c^
**Women (n = 257; 39.5% completion rate)**				
Weight, lb	251	196.0 (173.4 to 227.4)	251	−15.3 (−28.6 to −6.3)
Weight, %	NA	NA	251	−7.1 (−12.5 to −3.1)
BMI, kg/m^2^	251	32.7 (29.5 to 38.2)	251	−2.6 (−4.8 to −1.1)
Systolic blood pressure, mm Hg	199	123.8 (12.7)^d^	197	−5.3 (17.0)^d^
Diastolic blood pressure, mm Hg	199	75.7 (8.8)^d^	197	−6.3 (11.2)^d^
Body fat, %	216	42.5 (6.0)^d^	188	−2.7 (4.5)^d^
Waist, in	193	38.0 (35.4 to 41.9)	113	−4.0 (−6.4 to 2.0)
**Men (n = 91; 51.4% completion rate)**				
Weight, lb	85	248.5 (217.5 to 289.2)	85	−14.8 (−36.3 to −5.2)
Weight, %	NA	NA	85	−5.4 (−12.9 to −1.8)
BMI, kg/m^2^	85	34.6 (30.1 to 39.8)	85	−2.1 (−5.1 to −0.7)
Systolic blood pressure, mm Hg	67	125.7 (14.1)^d^	65	−7.1 (14.7)^d^
Diastolic blood pressure, mm Hg	67	76.6 (10.8)^d^	65	−6.4 (10.4)^d^
Body fat, %	64	33.2 (6.8)^d^	56	−2.9 (6.6)^d^
Waist, in	60	43.3 (38.8 to 50.0)	37	−3.0 (−5.1 to −1.6)

Abbreviations: IQR, interquartile range; NA, not applicable; BMI, body mass index.

a 762 participants could have completed phase II.

b Sample sizes vary because of missing data for some measures; negative change values indicate improvements from baseline to the end of phase II (1 year).

c Median and IQR are presented for data with skewed distributions unless otherwise noted.

d Measurement has a normal distribution and is presented as mean (standard deviation).

## Appendix. RE-AIM Components, Indices, and Calculation Equations

**Table:**

Component	RE-AIM Index	Calculation Equation
Individual participation rate (IPR)		No. of participants / no. of adult insurance agency members * (state's adult obesity prevalence rate + [state's adult overweight prevalence rate * overweight with at least 1 comorbid condition national prevalence rate])
Demographic representativeness (DR)		Mean ES (φ_c_^2^) of sex- and age-bracket χ^2^ comparisons of program participants and state residents
	*Reach (R)*	R = (IPR * [1 – DR]) * 100
Phase I individual completion rate (ICR_PhI_)	* *	No. of phase I completers / no. participants who could have completed phase I
Phase I outcome (O_PhI_)	* *	ES (η^2^) from 1-way RM ANOVA (IV: *time*; DV: *weight among phase I completers*)
Phase I differential impact (DI_PhI_)	* *	Mean ES (η^2^) from two 2-way RM ANOVAs: 1) IVs: *time* x *age bracket*; DV: *weight among phase I completers*, and 2) IVs: *time* x *sex*; DV: *weight* *among* *phase I completers*
	*Effectiveness (E)*	E = (ICR_PhI_ * O_PhI_ * [1 – DI_PhI_]) * 100
Setting participation rate (SPR)	* *	No. of active sites / (no. of cardiac rehabilitation centers + no. of physical therapy clinics + no. of hospitals + no. of wellness/fitness centers + no. of health clubs in the state)
	*Adoption (A)*	A = SPR * 100
Component implementation rate (CIR)	* *	Mean no. of "yes" responses to "Program Components" section of site survey / 14
	*Implementation (I)*	I = CIR * 100
Phase II individual completion rate (ICR_PhII_)	* *	No. of phase II completers / no. of participants who could have completed phase II
Phase II outcome (O_PhII_)	* *	ES (η^2^) from 1-way RM ANOVA (IV: *time*; DV: *weight among phase II completers*)
Phase II differential impact (DI_PhII_)	* *	Mean ES (η^2^) from two 2-way RM ANOVAs: 1) IVs: *time* x *age bracket*; DV: *weight among phase II completers*, and 2) IVs: *time* x *sex*; DV: *weight among phase II completers*
	*Maintenance: individual (M_I_)*	M_I_ = (ICR_PhII_ * O_PhII_ * [1 – DI_PhII_]) * 100
Setting continuation rate (SCR)	* *	No. of active sites that have continued to offer the program for 1 year or longer / no. of sites that started offering the program 1 year or more before study
	*Maintenance: setting (M_S_)*	M_S_ = SCR * 100

Abbreviations: ES, effect size; φ_c_^2^, squared Cramer phi; η^2^, partial eta squared; RM ANOVA, repeated measures analysis of variance; IV, independent variable; DV, dependent variable.
